# Pharyngeal Rhinosporidiosis

**DOI:** 10.4269/ajtmh.18-0903

**Published:** 2019-03

**Authors:** Satvinder Singh Bakshi

**Affiliations:** Department of Ear, Nose, and Throat, Head and Neck Surgery, Mahatma Gandhi Medical College and Research Institute (MGMCRI), Sri Balaji Vidyapeeth, Pondicherry, India

**Figure 1.**
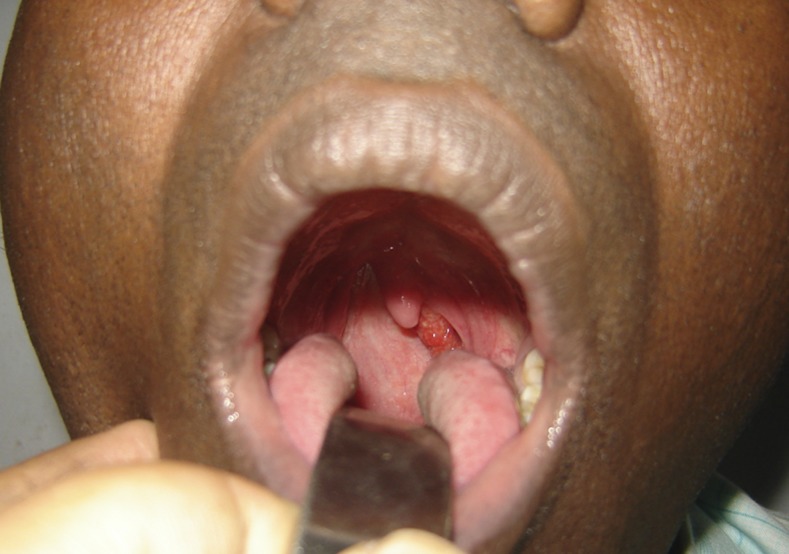
Patient with left-sided reddish nasal mass with white spots seen behind the posterior faucial pillar. This figure appears in color at www.ajtmh.org.

**Figure 2.**
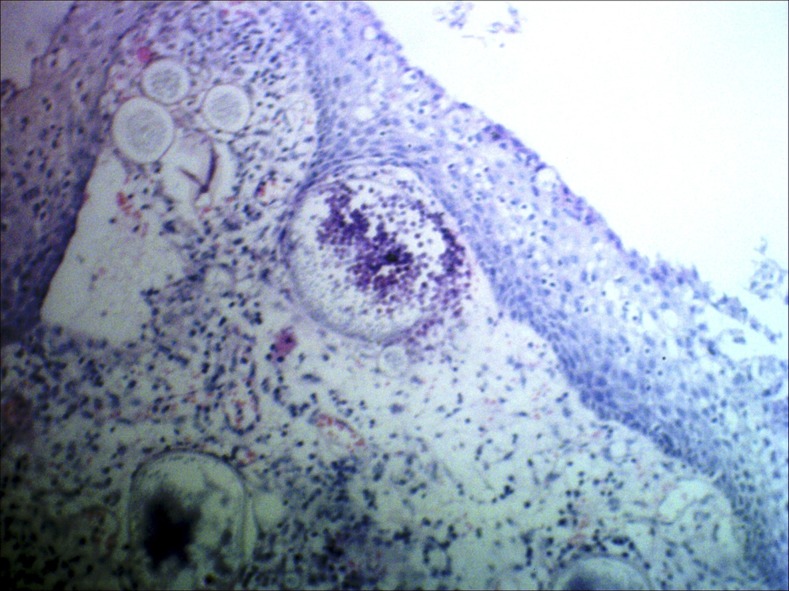
Subepithelium containing sporangia at various stages of development surrounded by dense mixed inflammation (HE, 100×). This figure appears in color at www.ajtmh.org.

A 52-year-old man presented with recurrent throat clearing and irritation since 2 months associated with occasional blood-tinged saliva. He was a known case of nasal rhinosporidiosis and had been operated 2 years ago. Examination revealed a reddish pedunculated fleshy mass with whitish spots posterior to the faucial pillar (Figure 1). Diagnostic nasal endoscopy revealed the mass to be attached from the lateral wall of the nasopharynx. Complete excision of the mass along with cauterization of the base with electric diathermy was carried out under general anesthesia. The postoperative biopsy revealed respiratory epithelium with multiple sporangia in the subepithelium surrounded by dense inflammation, confirmatory of rhinosporidiosis (Figure 2). Rhinosporidiosis is a chronic granulomatous disease caused by *Rhinosporidium seeberi*, which is classified under Mesomycetozoea.^1^ The disease is endemic in India, Sri Lanka, and tropical areas of Africa and South America.^2^ Most commonly it affects the nose, although mucous membrane of the nasopharynx, oropharynx, conjunctiva, rectum, and external genitalia can also be involved.^3^ Transmission occurs by bathing in stagnant ponds where animals also bathe. Patients usually present with a reddish nasal mass which bleeds profusely.^1,2^ The diagnosis is confirmed with excision biopsy of the lesion showing the typical sporangia in the tissue. The treatment is surgical excision followed by cauterization of the base to prevent recurrence. Dapsone has been tried in few cases to reduce the recurrence rate. Most patients require multiple surgeries as the recurrence rate is high.^2,3^
